# Automatic anxiety recognition method based on microblog text analysis

**DOI:** 10.3389/fpubh.2023.1080013

**Published:** 2023-03-20

**Authors:** Yang Yu, Qi Li, Xiaoqian Liu

**Affiliations:** ^1^Institute of Psychology, Chinese Academy of Sciences, Beijing, China; ^2^Learning and Cognition Key Laboratory of Beijing, School of Psychology, Capital Normal University, Beijing, China; ^3^Department of Psychology, University of Chinese Academy of Sciences, Beijing, China

**Keywords:** anxiety recognition, machine learning, social media platform, Weibo data, SC-LIWC

## Abstract

Mental health has traditionally been assessed using a self-report questionnaire. Although this approach produces accurate results, it has the disadvantage of being labor-intense and time-consuming. This study aimed to extract original text information published by users on the social media platform (Sina Weibo). A machine learning method was used to train the model and predict the anxiety state of the user automatically. Data of 1,039 users were collected. First, Weibo users were invited to fill the anxiety self-assessment scale. All original text data ever published by the users were collected. Second, the Simplified Chinese-Linguistic Inquiry and Word Count (SC-LIWC) were extracted for feature selection and model training. We found that the model achieved the best performance when the XGBoostRegressor algorithm was used. The Pearson correlation coefficient between the model predicted scores and self-reported scores was moderate (*r* = 0.322). In addition, we tested the reliability of the model, and found that the model had high reliability (*r* = 0.72). The experimental results further showed that the model was feasible and effective and could use the digital footprints to predict psychological characteristics.

## 1. Introduction

Anxiety (1) is a complex psychological state characterized by tension, uneasiness, apprehension, and other unpleasant feelings in response to an impending, potentially dangerous or threatening situation. Anxiety is also one of the most common emotional states. It is considered a positive stress instinct, and moderate anxiety creates excitement in the human brain, rapid thinking and reaction, which helps an individual to cope with an upcoming situation, which produces positive outcomes. Other scholars have shown that anxiety can interfere with work and sleep, and if it persists for long, it can cause anxiety disorders. In terms of manifestation, anxiety disorders typically present with “excessive and persistent fear and worry,” which impart serious negative effects on physical and mental health (2). For example, it affects the nervous system and causes sympathetic excitation, leading to vasoconstriction, increased blood pressure and heart rate, to induce cardiovascular diseases; severe anxiety disorders can even trigger depression (3), with the potential to cause self-injury or even suicide. Therefore, to prevent anxiety progression into anxiety disorders, effective strategies are needed to screen and track people with high anxiety status.

Traditionally, anxiety states have been assessed using self-report inventory (SRI). Although the final questionnaire score obtained by completing the Self-Rating Anxiety Scale (SAS) questionnaire can accurately measure the subject’s anxiety level (4), it has many shortcomings. For example, once participants have completed the questionnaire, the total score of the results need to be calculated manually. However, if the sample size of the measured population is too large, it will take several days to complete the calculation (5). If a scale is used to track a particular characteristic of a subject, the individual will be required to fill the same scale multiple times, which makes the individual to memorize the answers, leading to practice effect which reduces accuracy of the questionnaire. The time-consuming and labor-intensive nature of this method is not conducive for application in large populations.

Information technology has promoted the development of social media platforms, which have provided an ecosystem where people spend time in search for information and share their opinions and document their daily lives. Social media platforms are used to record the reactions, opinions and mental health levels of social users. Users express their moods and opinions in social media platforms by sharing different forms of messages such as text, images, videos, and emojis. Therefore, these digital footprints are potentially linked to individual behavioral and psychological characteristics (6). I In recent years, several researchers have used machine learning methods to predict personality traits based on what users post in social software (7–9) and other psychological traits (10, 11), such as anxiety disorders and depression, with good results.

In the study by Schwartz et al. (12), the content posted by 28,749 users on Twitter was extracted to build a regression model for predicting the users’ depression levels. Gruda and Hasan (13) used machine learning tools to construct a model for predicting the users’ anxiety levels based on their posts on Twitter. Kosinski et al. (14) studied a dataset comprising 58,000 Facebook users and used the content shared by these users on Facebook to develop a model that correctly distinguished between homosexual men and heterosexual men. Numerous studies have shown that machine learning can accurately predict personal attributes such as personality, anxiety, depression, and sexual orientation based on the digital footprints of users’ behaviors recorded in social media platforms. This non-invasive method can overcome the disadvantages of time-consuming and high labor costs of the traditional questionnaire assessment. It will also provide faster prediction of psychological indicators of large samples of people.

Sina Weibo, one of the most popular social networking platforms in China, is a micro-blog that allows users to easily post and access messages on the Web (15). Users can post texts, pictures, or short videos *via* World Wide Web (WEB), Wireless Application Protocol (WAP), and other approaches. For these reasons, many Chinese web users use it. According to official statistics, Weibo has 511 million monthly active users and 224 million daily active users as of September 2020. Therefore, the Sina Weibo contains the digital footprint of a large number of Chinese users.

Based on the discussion above, to overcome the shortcomings of traditional questionnaire measurements, we developed a model using machine learning methods for predicting users’ anxiety status based on the text content posted in Sina Weibo, and tested the performance of the constructed model.

## 2. Materials and methods

### 2.1. Data collection

Users on the Sina Weibo platform were recruited in October 2020 and invited users were asked to fill out the Chinese version of the Self-Rating Anxiety Scale (SAS) online. The SAS scale was developed by Zung (16) in 1971 as a self-report assessment tool for measuring people’s anxiety status. The Chinese version of the scale was employed in this study, and its validity and reliability were fully validated in previous studies. The SAS contains 20 multiple-choice questions and uses a 4-point scale, which mainly rates the frequency of symptoms defined by the items. The criteria were “1” no or very little time; “2” little time; “3” quite a lot of time; and “4 “most or all of the time. The main statistical indicator of SAS was the total score, range from 0 to 100, which can be simply interpreted as a higher total score representing a greater level of anxiety.

The users also filled in their demographic information, including the location, Weibo nickname, age, gender, and education level. Finally, 1,460 subjects were recruited and the completed questionnaires and information were collected. With permission from the subjects, the Python-based crawler technique was utilized to download their original tweets published publicly in the previous 60 days prior to filling out the questionnaire on the Sina Weibo Open Platform API,^1^ which contained about 189,000 entries.

### 2.2. Data preprocessing

To identify active Weibo users, information of users who posted less than 10 tweets in the past 60 days was excluded.

**Figure 1:**
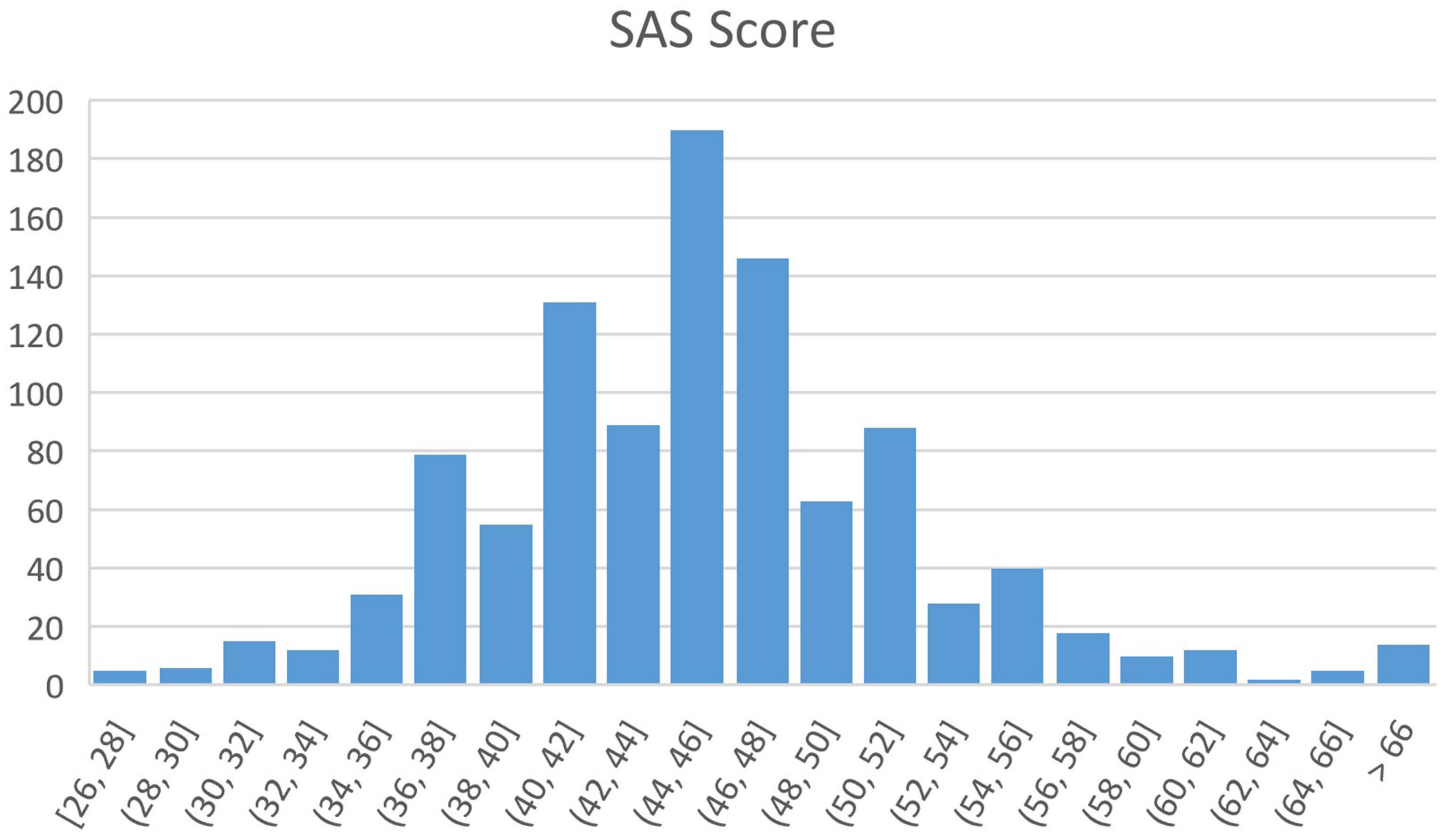
The distribution of SAS score.

After preprocessing, the final number of valid users was 1,039, including 495 males and 544 females, aged 18–56 years (*M* = 28.91, *SD* = 6.60). The demographic information of the subjects is shown in Table 1. The scores of anxiety self-assessment scale were in the range of 28–72 (*M* = 45.70, *SD* = 6.86) and the distribution of the scores is presented in Figure 1.

**Table 1:** Demographic information of participants.

Variable	Sample size (Frequency, %)
Total	1,039 (100.0)
Gender	
Male	495 (47.6)
Female	544 (52.4)
Age	
18–30	689 (66.3)
31–45	323 (31.3)
46–59	27 (2.4)
Education background	
High school or lower	38 (3.7)
College/technical school	234 (22.5)
University Bachelor school	740 (71.2)
Master’s degree or higher	27 (2.6)
Career background	
Civil servant	347 (33.4)
Manger/office clerk	154 (14.8)
Factory work/Agricultural worker	247 (23.8)
Business and service personnel	166 (16.0)
Other	125 (12.0)

### 2.3. Feature extraction

Initially, emoticons, pictures and videos were removed in the content published by users, to obtain text information, numbers and punctuation marks only. Next, the Simplified Chinese-Linguistic Inquiry and Word Count (SC-LIWC) dictionary was used to count the number of occurrences of each type of words. SC-LIWC is an effective dictionary for textual analysis of microblogs (17). It can calculate the percentage of multiple psychologically or linguistically meaningful words, which include words of emotional, cognitive, and social dimensions. Nan et al. (18) demonstrated the effectiveness of SC-LIWC in identifying psychological features. A total of 103 dimensions, including features such as the total number of words, the average number of words per sentence, and the dictionary coverage of the original tweet text posted by each user. Representative Chinese vocabulary examples for each emotion-related SC-LIWC category are shown in Table 2.

**Table 2:** Examples of Chinese words for each SC-LIWC category.

SC-LIWC category	Example words
Ver	分享、开车、听
We	我们、我俩、咱们
Tensem	已经、之前、日后
Sad	心痛、沮丧、无力
Excl	取消、但是、除外

### 2.4. Feature selection

In the machine learning process, if the features have too many dimensions, that problem of overfitting in the model training may occur, and overfitting means that the model has high prediction accuracy on the training set but low prediction accuracy on the test set, which decreases the generalization ability of the model. Therefore, before model training, feature selection needs to be performed on the feature files extracted in the previous step (19) to eliminate the features with high similarity. Thus, we first calculated the correlation coefficients between all features and counted the number of times that the correlation coefficient between each feature and other features exceeding 0.4. We set following criterion: if the number of times a feature showed high correlation with other features was greater than 30, then we consider these features to be redundant data and exclude them from the study. Subsequently, the Random Forest (RF) algorithm (20) was used to calculate the relative importance of the feature variables and rank the feature variables. Finally, the top 22 ranked dimensions were retained. Dimensional information is shown in Table 3.

**Table 3:** Examples of retain SC-LIWC category.

SC-LIWC category
Social
Work
Achieve
Money
Leisure
Affect
Negemo
Anx
Sad
Percept
Feel
Body
Health
Psychology
I
Motion
Discrep

### 2.5. Model training

The Self-Assessment Scale score for each user was used as the variable on label (*y*), and the 22 dimensions of information after feature selection were used as the feature matrix (*x*). A number of different algorithms were used to construct the model on the training set and evaluate the performance of the model on the test set. Since the label values were continuous data, we choose regression algorithms to adjust the parameters. Four common regression algorithms, including Linear Regression (LR), Support Vector Regression (SVR), XGBoostRegressor, and AdaBoostRegressor were employed. The model training process was conducted in Python 3.0.

### 2.6. Result

During the model training process, four algorithms used to adjust the parameters to achieve optimal model performance. A five-fold cross-validation and Pearson correlation coefficient determination were conducted between the model predicted SAS scores and the subjects’ actual SAS scores to assess the accuracy of the model predictions. The results of the models obtained using the four algorithms are shown in Table 4. It can be inferred that the model had the best prediction performance when the XGBoostRegressor algorithm was used compared with other algorithms, and the correlation coefficient between the model-predicted SAS scores and the subjects’ actual scores was more than 0.3 with a moderate correlation, the lowest mean absolute error (MAE) value.

**Table 4:** Results of the model.

Model	Pearson	Mean absolute error
LR	0.108	29.58
SVR	0.235	14.31
AdaBoostRegressor	0.287	9.89
**XGBoostRegressor**	**0.322**	**4.57**

Bold values mean that the model with the best results among the four models.

### 2.7. Split-half reliability of the model

Reliability is an important index in the assessment of consistency, stability, and reliability of training models, whereas split-half reliability is a common method for evaluating the internal consistency of training models ((21)). In this study, the odd-even split-half reliability was determined to test the reliability of the model. All original tweets of each user were collected and sorted based on the time of publication. The tweets were sub-divided into two datasets in odd and even positions. After completion of the model training, the two datasets were input into the model to obtain two sets of predictions, and the consistency between these two sets of predictions was calculated. In addition, the odd-even split-half reliability of the four models was calculated, and the results are presented in Table 5. It can be observed that the split-half confidence of the model reached 0.72 when the XGBoostRegressor algorithm is used, indicating that the training model was reliable.

**Table 5:** Comparison of the reliability results of the four models.

Model	Pearson
LR	0.68
SVR	0.67
AdaBoostRegressor	0.70
**XGBoostRegressor**	**0.72**

Bold values mean that the model with the best results among the four models.

## 3. Discussion

This study aimed to develop a model for predicting anxiety levels among Sina Weibo users based on text data. The features of all original posts published by microblog users were extracted as the input, the scores of anxiety self-assessment scales filled out by users in real life were used as the output. Four regression algorithms were selected to train the model. Finally, the performance of the model was verified by calculating the Pearson correlation coefficient and split-half reliability between the predicted scores of the model and the actual scores of the subjects. It was found that the constructed model by machine learning method could successfully predict the anxiety level based on data obtained from Weibo, and the highest Pearson correlation coefficient and split-half reliability of the model are achieved when the XGBoostRegressor algorithm was used.

Further analysis of the SC-LIWC word categories after feature selection showed that some word categories related to emotions were negative, such as NegEmo, Sad, Anx, and Discrep. This is consistent with findings from previous studies (22). Anxious people tend to express negative emotions in social media more than normal people. In addition, there are some word categories related to daily work and life, such as Work, Money, Leisure, Social, and Achieve. Our explanation for this is that in today’s society, work, social and money are considered important causes of anxiety, thus people with high anxiety are more likely to share their work and life content on the social media.

Anxious people use first-person singular pronouns (I) more frequently, whereas normal people often use third-person and first-person plural pronouns, which is consistent with previous studies (23). Higher anxiety can negatively affect physical and mental health (24), by inducing several conditions such as worry, avoidance, fear, sweating, nausea, and insomnia, and users with high anxiety tend to share these symptoms on the social media platforms. Therefore, word categories Health, Body, Feel, etc. related to body and mind are important features for constructing the model.

In terms of feature selection, we mainly used the Random Forests in this study (20). In most studies, the random forest is used method for feature selection (25, 26), because it can calculate the relative importance of feature variables using the out-of-bag (OOB) error and rank and filter feature variables, which makes it suitable when a large number of features are used to train the model. We tried to select the top 20 to 30 dimensions in the importance ranking and train the model with these features. Results showed that the model achieved the best results when the features in the top 22 dimensions were selected, hence we chose features among the top 22 dimensions in importance ranking as the final features for model training.

Our results revealed that the model achieved good performance when the XGBoostRegressor algorithm was used. The XGBoostRegressor algorithm is one of the XGBoost algorithms, a scalable Tree boosting algorithm, that is widely used in data science to obtain optimal solutions in many scenarios (27). In a previous study (28), the XGBoost algorithm was shown to successfully predict anxiety. In this study, we have further demonstrated that the XGBoost algorithm could effectively predict anxiety.

In previous studies, many classification algorithms have been employed in the construction of prediction models for predicting anxiety levels (29, 30), i.e., the output labels are discrete data. In contrast, in the present study, the constructed model outputs specific anxiety scores and validates the validity and reliability of the anxiety prediction model.

There are two limitations to this study. First, there are many subtypes of anxiety disorders (31), such as Generalized Anxiety Disorder (GAD), Panic Disorder (PD), agoraphobia, Social Anxiety Disorder (SAD) and Posttraumatic stress disorder (PTSD). Therefore, using the SAS, which is a simple tool for analyzing patients’ subjective symptoms, can only determine a person’s level of anxiety at the macro level, not the specific types of anxiety. Second, the emojis and pictures posted by the user also contain carry messages from the user (32). However, we only analyzed the plain text posted by users and did not include additional information in the analysis.

## 4. Conclusion

In this study, we developed a prediction model for assessing anxiety levels based on content posted by users on Sina Weibo text data using the Self-Assessment Scale for Anxiety (SAS). The model showed high reliability and validity. In addition, we demonstrated the feasibility and validity of users’ digital footprints on the platform to predict their psychological characteristics.

This study provides researchers and social media platforms with a low-cost, convenient and fast way to screen people with high anxiety. It lays the foundation for developing timely interventions to prevent psychological disorders. For example, the model constructed in this study can be applied to various social media platforms. By simply extracting the text content of users’ public posts in the background and inputting them into the constructed model, we can obtain the user’s anxiety score, identify people with high anxiety, and then alert them through private messages and provide professional help. This approach will help users to know their anxiety status in time and seek interventions in a timely manner. It should be noted that the output SAS scores of the models involved in this study can only be used as a reference and not as a professional diagnosis of anxiety disorders.

## Data availability statement

The data analyzed in this study is subject to the following licenses/restrictions: the datasets generated for this article are not readily available because the raw data cannot be made public. If necessary, we can provide model. Requests to access the datasets should be directed to the corresponding author. Requests to access these datasets should be directed to Anxiety recognition model, 852837384@qq.com.

## Ethics statement

Studies involving human participants were reviewed and approved by the scientific re-search ethics committee of the Chinese Academy of Sciences Institute of Psychology (H15010). The patients/participants provided written informed consent to participate in this study. Written informed consent was obtained from individual(s) for the publication of any potentially identifiable images or data included in this article.

## Author contributions

XL conceived the study and collected essential data for the study. QL and YY jointly designed the experimental flow of this study. YY developed the tools needed for the experiment, executed the whole experiment process, performed all statistical analyses, and wrote the manuscript with input from all authors. All authors contributed to the article and approved the submitted version.

## Conflict of interest

The authors declare that the research was conducted in the absence of any commercial or financial relationships that could be construed as a potential conflict of interest.

## Publisher’s note

All claims expressed in this article are solely those of the authors and do not necessarily represent those of their affiliated organizations, or those of the publisher, the editors and the reviewers. Any product that may be evaluated in this article, or claim that may be made by its manufacturer, is not guaranteed or endorsed by the publisher.

## Footnotes

^1^ http://open.weibo.com/
